# Black and White: Modified Black-Bottom Containers for Mass Egg Collection of *Wolbachia*-Infected *Aedes aegypti*

**DOI:** 10.3390/insects17040387

**Published:** 2026-04-02

**Authors:** Jonathan Wee Kent Liew, Muhammad Nashrul Suasni, Kee-Kee Chng, Chee-Seng Chong

**Affiliations:** Environmental Health Institute, National Environment Agency, 5008 Ang Mo Kio Ave 5, #06-13 Techplace II, Singapore 569874, Singapore; muhd_nashrul_suasni@nea.gov.sg (M.N.S.); chng_kee_kee@nea.gov.sg (K.-K.C.); chong_chee_seng@nea.gov.sg (C.-S.C.)

**Keywords:** *Aedes*, mass rearing, egg yield, colonization, mosquito release program

## Abstract

Efficient egg collection is important for large-scale mosquito rearing, but it presents logistical challenges and often yields suboptimal egg numbers in mass rearing cages. Furthermore, uneven distribution of eggs on oviposition substrates can lead to higher fatality of the first instar (L1) larvae among overlapping/stacked eggs. Previous studies showed that incorporating darker hues into oviposition pots (ovipots) improves egg collection, yet few discussed designs of containers for use in mass rearing cages. Using a no-choice experimental setup, the number of eggs collected per female was determined in cages of *Wolbachia*-infected *Aedes aegypti* mosquitoes provided with different ovipots. The percentage of dead larvae among the stacked eggs was also determined. Based on this information, different prototypes of a more efficient, larger ovipot were evaluated in mass rearing cages. A black-bottom container with white crepe paper collected the most eggs per blood-fed female by promoting higher oviposition rates. A significantly lower percentage (0.31%) of dead L1 larvae were found among relatively sparser eggs compared to 0.96% among stacked eggs. A larger, black-bottom ovipot with oviposition papers positioned at the center of the container, demonstrated suitability for use in mass rearing cages, promoting better egg distribution and yield.

## Simple Summary

Mosquito control programs that release modified mosquitoes to fight diseases like dengue fever need to produce millions of mosquito eggs. However, getting female mosquitoes to lay enough eggs in their large cages is challenging. Too many eggs concentrated on one spot could lead to high larvae fatality during hatching. This study tested different egg collection containers to address these challenges by comparing the egg numbers and hatch rates in various container designs, colors, and paper types. We found that containers with black-bottoms and white textured paper collected the most eggs, likely because mosquitoes are attracted to dark colors and prefer laying eggs on materials that hold moisture well. We also observed that adding more egg-laying papers within larger containers can provide additional surface area and prevent egg overcrowding, therefore reducing larval deaths while improving overall egg yield and collection efficiency. These improvements in egg collection methods will help facilities produce healthy mosquitoes more efficiently, making mosquito control programs more cost-effective.

## 1. Introduction

Mosquito rearing has been integral to the study of entomology and vector control. In recent years, innovative dengue control strategies such as *Wolbachia* [1,2,3] and the sterile insect technique (SIT) [4,5,6,7] have driven the transition from small-scale laboratory colonization to large-scale mass rearing. These approaches fundamentally require the mass production of millions of mosquito eggs in specialized facilities as the foundation for subsequent mosquito releases into the wild.

The mass rearing of *Aedes aegypti* faces significant challenges, notably in collecting sufficient eggs from female mosquitoes in large-scale rearing cages. Intriguingly, the egg yield per female in these cages is often lower than that observed under small-scale rearing conditions, even when the number of egg collection containers is increased. This discrepancy may be attributed to the skip oviposition behavior of *A. aegypti*, including their ability to withhold eggs for up to 7–10 days after blood feeding [8,9].

The selection of an oviposition site by gravid *Aedes* mosquitoes is a complex, multimodal process influenced by environmental and biological stimuli. Previous studies indicate that mosquito species use multiple sensory modalities—tactile, gustatory, olfactory, and visual cues—to evaluate oviposition sites based on characteristics such as color, reflectance, texture, moisture content, chemical composition, and biotic factors including conspecific density and microbial communities [10,11]. The presence of conspecific or heterospecific immatures can either attract (indicating suitable habitat) or deter (signaling resource competition) gravid females [11].

Among these stimuli, visual cues are critical factors enabling *Aedes* mosquitoes to identify suitable oviposition containers before physical substrate contact [11]. Research generally demonstrates a preference of *Aedes* mosquitoes for dark surfaces, though results can vary across studies. In laboratory studies, black oviposition containers were preferred by gravid *Aedes* mosquitoes over white ones [12], and collected the highest mean number of eggs compared to red, green, blue, and transparent containers [13]. However, there is also evidence to suggest that red surfaces can be equally or more attractive than black [14]. Williams and DeLong [15] also demonstrated in a no-choice test that using dark brown water in egg collection containers increased *A. aegypti* egg yield by 24% compared to clear water, accompanied by an increased oviposition rate.

Beyond simple color preferences, visual contrast significantly enhances attraction. McCrae [16] found that more *Anopheles* eggs were collected in black-backed containers against white cage floors compared to other color combinations, while Sippel and Brown [17] showed that mirroring surface, i.e., black enamel surface, was more attractive than a flat black surface.

However, these findings vary considerably across experimental conditions, species, and methodological approaches. Nonetheless, these suggest that altering the color, contrast, and reflectance of container components could potentially circumvent skip oviposition behavior and promote egg collection in mass rearing settings. Comprehensive studies on the design and evaluation of egg collection containers specifically for mass rearing cages should be carried out. Moreover, the significance of the egg collection process extends beyond quantity to the quality of collected eggs. Observations at the National Environment Agency (NEA)’s mosquito production facility in Singapore showed that female *A. aegypti* eggs were unevenly distributed among multiple egg collection containers within mass rearing cages, resulting in variable distribution and egg stacking. Consequently, we observed more dead first instar (L1) larvae among the stacked eggs upon hatching, highlighting the need for strategies that promote even egg distribution.

Therefore, using *Wolbachia* *w*AlbB-infected *A. aegypti* mosquitoes, this study aims to optimize egg collection efficiency in mass rearing facilities through four specific objectives: (i) evaluate egg yields from oviposition pots (ovipots) with different colored components; (ii) compare oviposition rates between black-bottom and clear ovipots; (iii) quantify larval mortality associated with stacked eggs upon hatching; and (iv) test prototype designs for more efficient egg collection in mass rearing cages. These findings can directly support large-scale mosquito release programs.

## 2. Materials and Methods

### 2.1. Mosquito Strain

The *Wolbachia*-infected *Aedes aegypti* strain, *w*AlbB-Sg, was used in this study. All experiments carried out with the adult mosquitoes were conducted in NEA’s mosquito production facility with environmental conditions set at 27.5 ± 1 °C, relative humidity of 80 ± 5% and a 12:12 h light:dark cycle.

### 2.2. Experimental Approach

This study employed a sequential experimental design, where findings from initial small-scale investigations informed subsequent evaluations. Experiments were conducted in two phases: first, using small experimental cages (30 × 30 × 30 cm) with fewer female mosquitoes to systematically compare different ovipot designs and components (Section 2.3 and Section 2.4); and second, applying the most promising ovipot designs in mass rearing cages housing approximately 7500–10,500 mosquitoes (Section 2.6). This iterative approach allowed us to optimize ovipot design parameters before scaling up to operational mass rearing conditions.

### 2.3. Comparing the Number of Eggs Collected by Different Ovipots

**Figure 1:**
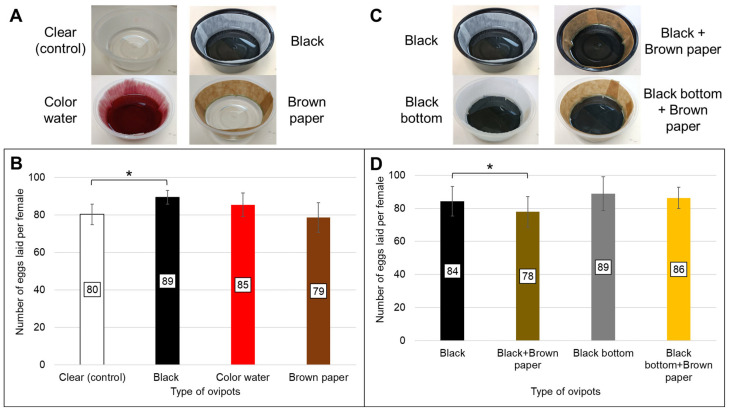
Ovipot variations and number of eggs laid per female in each ovipot. (**A**) Types of ovipots tested in the first experiment; (**B**) Average number of eggs collected from 3 blood meals over total number of blood-fed females alive to lay eggs after every blood meal (denoted by the numbers at the center of each bar); (**C**) Types of black and black-bottom ovipots tested in the second experiment; (**D**) Total number of eggs laid after the first blood meal over total number of blood-fed females alive to lay eggs after the blood meal. All error bars denote the standard deviations. The asterisk (*) denotes statistically significant differences between the groups, based on the linear mixed effects model. Color water ovipot: The water in the ovipot contains 1% *v*/*v* of each red and green food dye. Brown paper ovipot: Anchor seed germination paper is used as the oviposition paper. Black-bottom ovipot: Made by taping black duct tape at the bottom in the interior of the container.

This part of the investigation was carried out in two separate experimental runs, whereby four different ovipots were tested in each run (Figure 1). The containers were round, transparent or black polypropylene plastic bowls (385 mL capacity; 119 mm top diameter × 54 mm height) (SKP Pte Ltd., Singapore). Two types of oviposition papers were tested: (i) 76# heavy weight creped seed germination paper (Anchor Paper Co., Saint Paul, MN, USA), which is light brown in color, and (ii) creped, qualitative, technical paper of grade 39/N (Sartorius, Goettingen, Germany; item no.: FT-2-483-580580), which is white. These papers were cut into strips of 18 cm × 3.5 cm. The strips were cut in such a way that the ridges were oriented vertically when used. Due to inherent textural differences between both papers, the side of the creped qualitative paper, which is less “crumpled”, or smoother (but remains textured), was faced outwards for the mosquitoes to lay eggs on, while the side of the Anchor paper, which was deemed to be more “crumpled”, was used. This approach aimed to achieve comparable surface roughness between the two colored substrates. All ovipots contained 60 mL of reverse osmosis water, unless specified otherwise.

In the initial experiment, 20 blood-fed females from the same batch were aspirated and transferred into each 30 × 30 × 30 cm cage. Each cage received a different type of ovipot for testing: (a) clear ovipot with white, creped technical paper acting as control (this is the ovipot type routinely used in our rearing protocol); (b) black ovipot: black container lined with white, creped technical paper; (c) color water ovipot: clear container lined with white, creped technical paper and filled with water containing 1% *v*/*v* each of red and green food dyes; and (d) brown paper ovipot: clear container lined with Anchor seed germination paper (Figure 1A). Three cages (triplicates) received the same type of ovipot. The female mosquitoes were subsequently provided the 2nd and 3rd blood meals in their respective cages. Each cage received the same type of ovipot for egg collections after each blood meal. This investigation was performed 3 times. Additionally, rank-order correlation (Kendall’s rank correlation test) was used to assess whether the relative performance of different ovipot types remained consistent across 1, 2, and 3 blood meals. This was to determine whether a single blood meal is sufficient for evaluating ovipot efficacy.

The second experiment was a follow-up investigation based on the findings from the initial experiment. Four ovipot configurations were evaluated: (a) black ovipot; (b) black-bottom ovipot: made by taping black duct tape at the bottom in the interior of the clear container and lined with white, creped technical paper; (c) black + brown paper ovipot: black container lined with Anchor seed germination paper; and (d) black-bottom + brown paper: black-bottom container lined with Anchor seed germination paper (Figure 1C). Eggs were collected only after the first blood meal. The experiment was repeated three times.

The schedule of the blood meals, provision of ovipots and removal of ovipots from the cages can be found in Supplementary Table S1. Dead or moribund female mosquitoes were recorded daily to determine the number of blood-fed females which were alive to lay eggs after each blood meal. The ovipots were ranked based on the total number of eggs collected on the oviposition papers from 3 (or 1) blood meals over total number of blood-fed females alive to lay eggs after each blood meal.

### 2.4. Influence of Black-Bottom Ovipot on the Oviposition Rate of Female Mosquitoes

Based on the outcomes of the preceding experiments, the effects of using the clear (control) ovipot and black-bottom ovipot on the oviposition rates of gravid female mosquitoes were compared. Twenty blood-fed females from the same batch were aspirated and transferred into each 30 × 30 × 30 cm cage. Nine cages of mosquitoes were divided into groups of three, i.e., Group 1, 2 and 3. The black-bottom ovipot and clear ovipot were provided to Group 1 and 2, respectively. In Group 3, the two types of ovipot were alternated at intervals (see Supplementary Table S2). Ovipots were first provided to the cages 2 days after the blood meal. Ovipots in the respective cages were changed at every 6 or 12 h interval for a period of 48 h, after which the ovipots were left in the cages for three consecutive 24 h intervals—ovipots were present in the cages for a total of 120 h. The number of eggs collected at each interval was counted.

### 2.5. Obtaining Non-Stacked Eggs on the Oviposition Papers and Determining the Percentage of Dead L1 Larvae Among These Eggs

Among the few plausible ways to reduce stacking of eggs, i.e., by promoting a more even distribution of eggs, the water level in the ovipots was adjusted to facilitate egg deposition across the surface area of the oviposition paper. This exploits the tendency of the gravid females to lay eggs along the water line [18].

Two round ovipots (Ovipot A and Ovipot B) were placed in a cage with approximately 10,500 mosquitoes (1:2.5 male:female ratio) on the second day after their first blood meal. Both were left for 44 h in this cage. Ovipot A had a constant, lower water level (40 mL water), to collect stacked eggs along the water line. Ovipot B contained an initial high-water level (200 mL water). Then, at every 2–4 h during working hours, the water level in Ovipot B was reduced by pipetting 20–30 mL of water from the container each time.

At the end of the egg collection period, a 1–1.5 cm wide portion was cut from the center of two oviposition papers from each ovipot (2 cut portions from each ovipot). The number of eggs on each portion was estimated using ImageJ (version 1.53k). Then, these were stored at 23.5 ± 0.5 °C ang relative humidity of 80 ± 5% under a 12:12 h light:dark cycle.

Experiments were conducted in two independent temporal blocks, each containing two replicates per condition (*n* = 4 total replicates per condition). The eggs which had been stored for 2–3 weeks were hatched concurrently. The percentage of dead L1 larvae upon hatching against the estimated number of eggs on each portion was computed. A larva was confirmed to be dead when observed to be immobile and not attached to the eggshell. Dead larvae attached to eggshells were excluded, as mortality may have resulted from premature hatching.

### 2.6. Investigating the Distribution of Eggs Laid on the Oviposition Papers in Different Oviposition Containers in a Mass Rearing Cage

**Figure 2:**
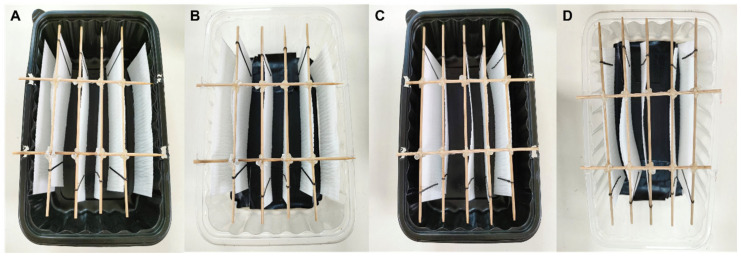
Different rectangular ovipots tested in the mass rearing cages: (**A**) black container with oviposition papers at the center and against the wall; (**B**) black-bottom container with oviposition papers at the center and against the wall; (**C**) black container with all oviposition papers at the center; and (**D**) black-bottom container with all oviposition papers at the center.

Based on the outcomes of the investigations above, a larger ovipot (225 mm length × 156 mm breadth × 69 mm height) was fabricated to be tested in the mass rearing cages with approximately 10,500 mosquitoes (1:2.5 male:female ratio). Two different types of frames to hold the oviposition paper strips were tested (Figure 2). In the container with oviposition papers (16 × 6 cm) at both center and against the wall, four pairs of oviposition paper strips were clipped back-to-back in the middle, while the remaining two strips were each placed against the container wall (Figure 2A,B). In the container with all oviposition papers at the center, five pairs of oviposition papers were clipped in the middle of the container (Figure 2C,D). The distance between each strip/pair of strips in the container was approximately 2–2.5 cm.

Two cages of blood-fed mosquitoes were each provided with a container having oviposition papers positioned at both center and walls, while two other cages each received a container with all oviposition papers placed only at the center. This experiment was repeated four times. Three repeats used full black containers throughout, while one repeat used black-bottom containers throughout. Within each repeat, identical container types were used in all cages. The number of eggs collected on each oviposition paper in each container was estimated using ImageJ.

For the subsequent test, we divided the cages into two groups. The first group received full black containers, while the second group received black-bottom containers. Each group consisted of three cages: two containing 10,500 mosquitoes (1:2.5 male:female ratio) and one containing 7500 mosquitoes (1:1 male:female ratio). All containers had oviposition papers positioned at the center. Data were collected continuously from all six cages across three blood meals. The number of eggs collected on each oviposition paper in each container was estimated using ImageJ.

Egg distribution uniformity was assessed using two measures: (1) standard deviation of egg counts across oviposition papers, where a lower value indicated more uniform distribution; and (2) the proportion of total eggs on each paper. With 10 oviposition papers per container, an ideal uniform distribution would result in each paper containing approximately 10% (or 0.1×) of the total eggs collected in each container.

### 2.7. Statistical Analysis

All data collection and analyses were performed in Microsoft Excel (Microsoft Office 365) or R (version 4.4.1). One-tailed t-Test and Levene’s test for equal variance were carried out using Microsoft Excel. Kendall’s rank correlation test was carried out using the cor.test function. Linear mixed models (LMM) were fitted with restricted maximum likelihood (REML), using the lmer and emmeans function to analyze the influence of different types of ovipots on number of eggs collected. The different types of ovipots (clear, black, color water, brown paper for first experimental run; black, black with brown paper, black bottom, and black bottom with brown paper for second experimental run) were treated as fixed effects, while experimental rounds and cages were treated as random effects. Significance of fixed effects was assessed using Satterthwaite’s method, and no extreme outliers were detected in the residuals. Median time to egg collection was determined using the survival and survminer R packages, with log-rank tests and Benjamini–Hochberg-adjusted pairwise comparisons to compare oviposition timing between groups. A linear mixed-effects model was also fitted to analyze the oviposition rate, with treatments (Group 1, 2, 3) as fixed effects, time as a continuous variable, and replicates of each treatment as random effects to account for cage variation.

## 3. Results

### 3.1. Significant Correlation Across Blood Meal Collections

Given the egg-retention behavior of *A. aegypti* female mosquitoes, we sought to determine the most effective cycle for oviposition data collection. Specifically, rank-order correlations were calculated to determine whether ovipot performance rankings remain consistent across successive blood meals, thereby allowing data collection to be limited to fewer gonotrophic cycles. Using data collected from the first experimental run, Kendall’s rank correlation analysis revealed significant correlations between the rank orders of the ovipots (i) after the first blood meal and (ii) after the first two blood meals, with that after the third blood meal (correlation coefficient Tau = 0.78, *p* = 0.002 for both comparisons). Based on these findings, it was concluded that collecting data after the first blood meal was sufficient to represent the overall oviposition outcomes of *A. aegypti* females in the current experimental setup.

### 3.2. Black-Bottom Ovipot Collected the Most Eggs per Blood-Fed Female Mosquito

To account for individual and cohort biological variation that may influence results, a linear mixed effects model (LMM) was fitted to assess the effect of different ovipot types on the number of eggs collected in both the first and second experimental runs. In the first experimental run, the overall effect of the different types of ovipots was significant [F_3,30_ = 6.22, *p* = 0.002]. Compared to clear ovipots that obtained an average of 80.32 eggs per blood-fed female (SE = 2.16), black ovipots obtained a significantly higher average of 89.42 eggs per blood-fed female (β = 9.10, SE = 2.77, t = 3.29, *p* = 0.003) (Figure 1B, Table 1). While the model showed that color water ovipots obtained a higher average of 85.38 eggs per blood-fed female (β = 5.06, SE = 2.77, t = 1.83, *p* = 0.078) and brown paper ovipots obtained a lower average of 78.71 eggs per blood-fed female (β = −1.61, SE = 2.77, t = −0.58, *p* = 0.565), neither effect was statistically significant.

Additionally, post hoc Tukey-adjusted pairwise comparisons revealed that black ovipots (estimated mean = 89.42) collected significantly more eggs when compared to both clear ovipots (estimated mean = 80.32, *p* = 0.015) and brown paper ovipots (estimated mean = 78.71, *p* = 0.004), while other pairs did not differ significantly (Supplementary Table S3).

In the second experimental run, eggs were collected and counted for the first blood meal. Similarly, the overall effect of the different types of ovipots used in this run was significant [F_3,24_ = 5.80, *p* = 0.004]. The black ovipot collected an average of 84.27 eggs per blood-fed female (SE = 4.42), while the black ovipot with brown paper collected a significantly lower average of 77.86 eggs per blood-fed female (β = −6.41, SE = 2.76, t = −2.32, *p* = 0.029) (Figure 1D). While black-bottom ovipots and black-bottom ovipots with brown paper collected a higher average of 88.84 eggs (β = 4.57, SE = 2.76, t = 1.65, *p* = 0.111) and 86.36 eggs (β = 2.09, SE = 2.76, t = 0.76, *p* = 0.457) per blood-fed female, respectively, when compared to the black ovipots, this difference was not significant.

Post hoc Tukey-adjusted pairwise comparisons for the second experimental run showed that black ovipots with brown paper (estimated mean = 77.86) collected significantly fewer eggs than both the black-bottom ovipots (estimated mean = 88.84, *p* = 0.003) and black-bottom ovipots with brown paper (estimated mean = 86.36, *p* = 0.025). All other pairwise comparisons were not statistically significant (Supplementary Table S3).

Overall, the combination of a black-bottom container lined with white, crepe technical paper strips collected the most eggs.

### 3.3. Black-Bottom Ovipot Collected Eggs at a Higher Rate Compared to Clear Container Ovipot

**Figure 3:**
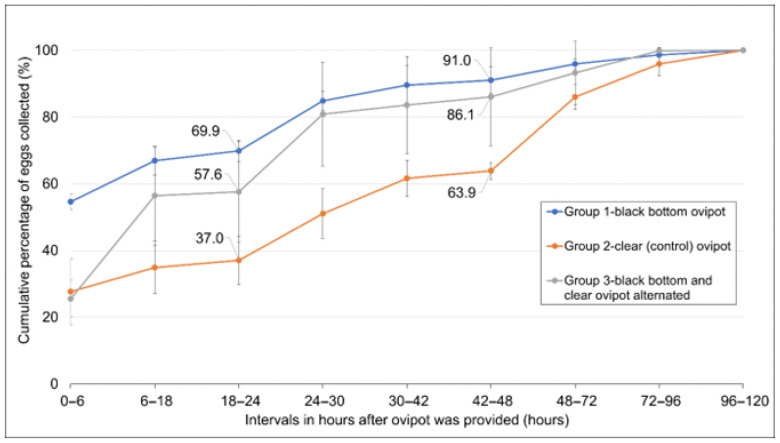
Average cumulative percentage of eggs collected over time when different ovipots were provided to the cages of mosquitoes. The error bar indicates standard deviations.

When black-bottom ovipots were used, a higher rate of egg collection was observed in comparison to cages provided with clear container ovipots (Figure 3). Within 48 h, about 91% of the eggs were collected by the black-bottom ovipot (Group 1), in contrast to the 64% of eggs collected by the clear (control) ovipot (Group 2) (Figure 3). When these two types of ovipots were alternately provided, provision of the black-bottom ovipots was observed to “rescue” the lower egg collection rate associated with the clear (control) ovipots, as evident at the 6–18 h and 24–30 h intervals. By the end of the 48 h period, 86% of the total eggs were collected (in Group 3) in this manner, which was higher than that when only the clear ovipots were used (Figure 3). The average number of eggs collected from Group 1, 2 and 3 were 1986, 1997 and 1892, respectively. These observations were consistent with the Kaplan–Meier analysis of egg collection over time, which revealed significant differences among the different ovipots in the log-rank test (χ^2^ = 1684, df = 2, *p* < 0.0001). Subsequently, pairwise comparisons with Benjamini–Hochberg adjustment showed that all Groups differed significantly (*p* < 0.0001) in their egg collection rate. Based on the median cumulative percentage of egg collected, Group 1 showed the fastest egg collection (6 h), followed by Group 3 (18 h) and Group 2 (30 h) (Figure 3).

In addition, oviposition rates across the entire oviposition period (0–120 h) were also fitted with LMM for analysis. The overall effect of the ovipot types on egg collection was found to be significant in an ANOVA F-test [F_2,69_ = 7.56, *p* = 0.001], indicating that the rate of egg collection was significantly different among Groups 1, 2 and 3. Post hoc Tukey-adjusted pairwise comparisons revealed that the average cumulative egg collection across 120 h was significantly higher for black-bottom ovipots (Group 1, 83.5 ± 3.53% SE) compared to clear control ovipots (Group 2, 62.0 ± 3.53% SE) (*p* = 0.012). While alternating ovipots (Group 3, 75.9 ± 3.53% SE) collected a higher proportion of eggs across 120 h when compared to clear ovipots (Group 2), no significant difference was observed (*p* = 0.071). No significant difference was observed between black-bottom (Group 1) and alternating ovipots (Group 3) (*p* = 0.350).

### 3.4. Significantly More Dead Larvae Were Found Among Stacked Eggs

By adjusting the water levels in the ovipots, eggs with little to no stacking were successfully obtained (see Supplementary Figure S1 online). A significantly higher percentage of dead larvae was found in areas where eggs were stacked, compared to those on paper strips with little to no stacking/overlapping of eggs [t(6) = 4.03, *p* = 0.003]. Additionally, 0.96% larvae (SD = 0.31) were found dead among the stacked eggs (estimated number of eggs on each strip: 1268–2171) compared to 0.31% dead larvae (SD = 0.10) on the oviposition papers from Ovipot B, in which the water level was adjusted at intervals (estimated number of eggs on each strip: 1414–3358).

### 3.5. Black-Bottom Container with Oviposition Papers Positioned at the Center Collected Eggs Which Were More Evenly Distributed

**Figure 4:**
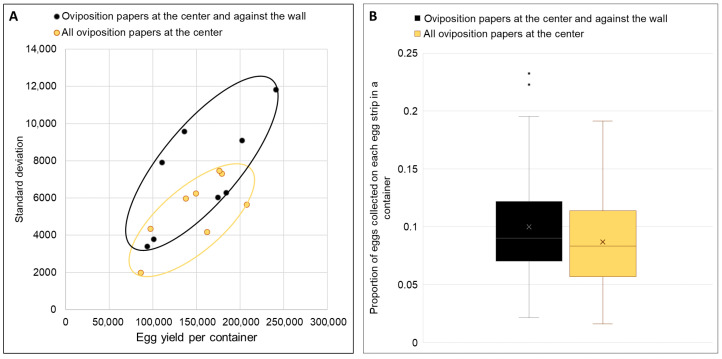
(**A**) Standard deviations of the number of eggs on each strip in the container with oviposition papers at the center and against the wall, and those in the container with all oviposition papers at the center, against the estimated total number of eggs collected in the respective container; (**B**) Box-plots showing proportion of eggs collected on each oviposition paper in a container either with oviposition papers at the center and against the wall or with all oviposition papers at the center of the container. The middle line in the box indicates the median, while the cross indicates the mean value.

The estimated number of eggs on each oviposition paper in the container with all oviposition papers at the center exhibited a slightly lower standard deviation than that of the container with oviposition papers at the center and against the wall (Figure 4A), indicating a relatively more even distribution of eggs within the former container. When the proportions of eggs collected on each egg strip within a container were aggregated, Levene’s test for equal variance showed no significant difference in variances between the two groups [F(1,170) = 1.61, *p* > 0.05]. Nonetheless, having all oviposition papers at the center of the container was determined to have a slight advantage over the other arrangement (Figure 4), in terms of the distribution of the eggs.

**Figure 5:**
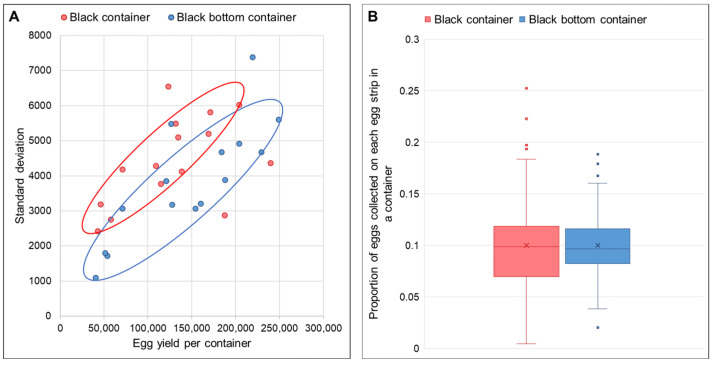
(**A**) Standard deviation of the number of eggs on each strip in the black and black-bottom container against the estimated total number of eggs collected in the respective container; (**B**) Box plots showing proportion of eggs collected on each oviposition paper in either a black or black-bottom container. The middle line in the box indicates the median, while the cross indicates the mean value.

Based on Figure 5, the estimated number of eggs on each oviposition paper in the black-bottom container exhibited a lower standard deviation than that of the black container, indicating a relatively more even distribution of eggs on the oviposition papers within the black-bottom container. Levene’s test for equal variance showed that the variances of the proportion of eggs on each egg strip between the two containers were significantly different, F(1,238) = 7.61, *p* < 0.01.

Furthermore, the black-bottom ovipots collected about ≥10% more eggs compared to the black container, and even more than that collected by the clear (control), round ovipots (see Supplementary Figure S2 online). This corroborates the results obtained from the small-scale investigations above.

## 4. Discussion

Efficient use of resources during large-scale mass rearing of mosquitoes necessitates maximizing egg production and usage. Previous studies have predominantly explored parameters such as adult densities, male:female ratio, conditioning of eggs and hatching, egg storage [19,20,21], number of ovipots to use and blood meal delivery methods [22], while others have enhanced egg collection by improving chemical cues [23,24]. The current study focuses on visual aspects, demonstrating that ovipot color and design significantly impact egg collection without requiring adjustments to mosquito densities or blood feeding frequency. Specifically, black-bottom containers increased total egg yield, accelerated oviposition rates, and promoted more uniform egg distribution when combined with centrally positioned oviposition papers, addressing critical bottlenecks in mosquito mass production.

It is important to note the methodological approach employed in the current study. No-choice tests rather than preference tests were adopted, as preference tests reveal relative attractiveness but may not predict absolute egg numbers—a mosquito may prefer one site without depositing most of its eggs there. No-choice tests are able to determine which ovipot collects the highest absolute number of eggs rather than measuring relative preferences. This approach also avoids confounding positional effects on oviposition site selection within cages, as observed by us and others [16]. This methodological difference should be considered when comparing our results with previous preference-based studies. Additionally, ovipot rankings after the first blood meal significantly correlate with rankings after multiple blood meals, indicating that single blood meal data sufficiently represent overall oviposition outcomes of *A. aegypti* females in the current experimental setup. This finding validates our approach and could enhance experimental efficiency in similar investigations, without sacrificing quality and reliability.

The better performance of black-bottom containers aligns with current understanding of mosquito oviposition behavior. Previous studies have demonstrated gravid mosquito preferences for black ovipots [12,13], black-backed ovipots against contrasting cage floors [16], and dyed oviposition water [15,25]. Our results extend these findings by showing that black-bottom containers collected more eggs per blood-fed female than either full black or clear containers (Figure 1). This enhanced collection likely results from attractive visual contrast at the container bottom, where mosquitoes rely on optical cues during oviposition [12,25]. McCrae [16] demonstrated that egg collection efficiency in *Anopheles gambiae* increases with background contrast intensity, supporting our observation that the black-bottom design creates optimal visual stimulation. In another related study, *A. aegypti* females were found to oviposit the highest number of eggs in black ovitrap followed by red ovitrap [26]. Nonetheless, our results with brown-colored water diverge from Williams and DeLong [15] and Beehler et al. [25], possibly reflecting differences in dye composition, egg-laying substrates, ovipot materials, or species-specific responses. Beehler et al. [25] found that dyed water was the strongest attractant for *A. triseriatus* (89% of eggs), overshadowing any container color effects.

Oviposition substrate characteristics proved equally important. White crepe paper collected more eggs than brown Anchor seed germination paper, contrasting with Panigrahi et al. [27], who reported preferences for black ovistrips. This apparent contradiction likely reflects the multifactorial nature of substrate selection, where surface texture [28] and moisture retention capacity [11] may outweigh color preferences. The white crepe paper (approximately 650 µm thick) likely retained more moisture than the thinner Anchor seed germination paper. Momen et al. [28] found that crepe brown seed germination papers of varying thicknesses differed in collection efficiency, and our unpublished tests with different white crepe papers confirm that physical properties influence egg collection independent of color.

Using black-bottom ovipots leads to significantly higher oviposition rates, collecting 91% of eggs within 48 h compared to 64% for clear containers (Figure 3). This acceleration addresses the egg-retention behavior characteristic of *A. aegypti* [8,9] and may enable more frequent collection cycles in production facilities. The mechanism likely involves visual contrast stimulating egg-laying or reducing egg-retention tendencies [29,30,31], though sugar availability may also lead to less motivation by the mosquitoes to lay eggs [32].

Beyond egg yield, achieving uniform egg distribution addresses a critical quality issue in mass production. *A. aegypti* females preferentially lay eggs along water lines [18], creating stacked eggs that exhibited significantly higher L1 larval mortality (0.96% versus 0.31% for dispersed eggs). While this percentage appears modest, it translates to 100,000 larvae lost per 10 million eggs hatched at production scale. While eggs can be brushed off the oviposition substrate, thereby eliminating the circumstances arising from hatching stacked eggs, the abrasion may damage the eggs, which is counterproductive. Further innovations are required to optimize egg collection methods and prevent egg stacking in mass rearing settings. While adjusting water levels successfully promoted even distribution, the absence of a practical solution for mass rearing settings led us to develop an interim approach. Centrally positioning oviposition papers in black-bottom containers achieved more uniform distribution (lower standard deviation and variance in egg counts per strip) while maintaining high total yields. This is consistent with results from the small-scale experiments. This design also capitalizes on the preference of *Aedes* mosquitoes for containers with larger surface areas [12,27] or size [33], as demonstrated by enhanced performance of our scaled-up rectangular containers. While our facility has also implemented this design for egg collection from *A. albopictus* with promising preliminary results, comprehensive validation of these findings across different *Aedes* species requires further investigations.

Therefore, the integration of black-bottom containers with centrally positioned oviposition papers into our facility’s mass rearing operations demonstrates practical viability. These design improvements address both quantity and quality without increasing operational complexity, contributing to more efficient production of *Wolbachia*-infected mosquitoes for vector control programs and ultimately supporting public health interventions against dengue and other arboviral diseases.

## 5. Conclusions

This study demonstrates that black-bottom containers significantly enhance egg collection in mass rearing settings, likely by stimulating increased oviposition rates among gravid female mosquitoes. Moreover, there was a higher mortality rate of L1 larvae in areas where eggs were densely stacked or overlapping, thus revealing a trade-off between quantity and viability. Our findings indicate that in large-scale mass rearing, the use of larger ovipots featuring centrally placed oviposition papers within black-bottom containers leads to both improved egg distribution and increased overall egg collection. This ovipot design is now currently used in our mosquito production facility. This research provides a comprehensive examination of visual cues and substrate characteristics influencing mosquito oviposition behavior. These findings contribute to the optimization of mass rearing techniques and expand our understanding of mosquito biology, potentially paving the way for innovative solutions in the field. Furthermore, the validation that single blood meal data adequately represent overall oviposition outcomes enhances experimental efficiency for future investigations.

## Figures and Tables

**Table 1:** Summary of Linear Mixed Models assessing the effects of different types of ovipots on the number of eggs laid per blood-fed female. The first experimental run compared clear (reference), brown paper, black, and color-water ovipots. The second experimental run evaluated variations of black ovipots: full black (reference), black + brown paper, black bottom, and black bottom + brown paper. Estimates (β) represent the difference in egg counts relative to the reference condition (Clear for first run, Black for second run), with positive estimates indicating an increase while negative estimates indicate a reduction in egg numbers.

	Treatment	Estimate (β)	Standard Error (SE)	t-Value	*p*-Value
First run of experiment	Intercept (Clear)	80.32	2.16	37.25	<0.0001
	Brown paper	−1.61	2.77	−0.58	0.565
	Black	9.10	2.77	3.29	0.003
	Color water	5.06	2.77	1.83	0.078
Second run of experiment	Intercept (Black)	84.27	4.42	19.06	0.0005
	Black + Brown paper	−6.41	2.76	−2.32	0.029
	Black bottom	4.57	2.76	1.65	0.111
	Black bottom + Brown paper	2.09	2.76	0.76	0.457

## Data Availability

The original contributions presented in this study are included in the article/Supplementary Materials. Further inquiries can be directed to the corresponding author.

## Supplementary Materials

The following supporting information can be downloaded at: https://www.mdpi.com/article/10.3390/insects17040387/s1. Table S1: Schedule of blood meals, and provision and removal of ovipots from cages; Table S2: Provision and change of ovipots in the different groups of cages at different time intervals; Table S3: Tukey-adjusted pairwise comparisons of ovipot types across first and second experimental runs. Pairwise comparison results show estimated mean differences in number of eggs per blood-fed female collected by different ovipot types, standard errors (SE), t-ratios, and adjusted *p*-values. Statistically significant differences (*p* < 0.05) are indicated with asterisks (*). Figure S1: Example of cut oviposition papers with stacked eggs (from Ovipot A) and those with little to no stacked eggs (from Ovipot B). The red arrows point to dead L1 larvae among the stacked eggs; Figure S2: (A) Total number of eggs collected across three blood meals from the cages (*n* = 1) with 7500 mosquitoes using different ovipots.; (B) Average number of eggs collected across three blood meals from cages with 10,500 mosquitoes (*n* = 2) using different ovipots.
