# Supplementary material for: Black and White: Modified Black-Bottom Containers for Mass Egg Collection of Wolbachia-Infected Aedes aegypti
Source: Insects. 2026 Apr 2;17(4):387. doi: 10.3390/insects17040387 (PMC13115564; doi:10.3390/insects17040387)
Supplement: Supplementary file 1 [file insects-17-00387-s001.zip › insects-4209934-supplementary.pdf]

Supplementary information

Supplementary Table S1. Schedule of blood meals, and provision and removal of ovipots from cages

Day(s) post 1st blood meal	Provision or removal of ovipots	Subsequent blood meal (if any)
1		
2	Ovipot#1 provided	
3		2nd blood meal
4	Ovipot#1 removed	
5	Ovipot#2 provided	
6		
7	Ovipot#2 removed	3rd blood meal
8		
9	Ovipot#3 provided	
10		
11	Ovipot#3 removed	

Supplementary Table S2. Provision and change of ovipots in the different groups of cages at different time intervals.

Interval in hours after ovipots were first introduced	0 - 6	6 - 18	18 - 24	24 - 30	30 - 42	42 - 48	48 - 120
Group 1	Black bottom ovipot						
Group 2	Clear ovipot						
Group 3	Clear	Black-bottom	Clear	Black-bottom	Clear	Black-bottom	Black-bottom

Supplementary Table S3. Tukey-adjusted pairwise comparisons of ovipots types across first and second experimental runs. Pairwise comparison results show estimated mean differences in number of eggs per blood-fed female collected by different ovipots types, standard errors (SE), t-ratios, and adjusted P-values. Statis-tically significant differences (P<0.05) are indicated with asterisks (*).

	Contrast	Estimate	Standard Error (SE)	df	t-ratio	P-value

First run of experiment	Clear – Brown paper	1.61	2.77	24	0.58	0.937
	Clear – Black	-9.10	2.77	24	-3.29	0.015*
	Clear – Color water	-5.06	2.77	24	-1.83	0.287
	Brown paper – Black	-10.71	2.77	24	-3.87	0.004*
	Brown paper – Color water	-6.67	2.77	24	-2.41	0.103
	Black – Color water	4.04	2.77	24	1.46	0.476
Second run of experiment	Black – (Black + Brown paper)	6.41	2.76	24	2.32	0.121
	Black – Black bottom	-4.57	2.76	24	-1.65	0.369
	Black – (Black bottom + Brown paper)	-2.09	2.76	24	-0.76	0.873
	(Black + Brown paper) – Black bottom	-10.98	2.76	24	-3.98	0.003*
	(Black + Brown paper) – (Black bottom + Brown paper)	-8.50	2.76	24	-3.08	0.025*
	Black bottom – (Black bottom + Brown paper)	2.48	2.76	24	0.90	0.806

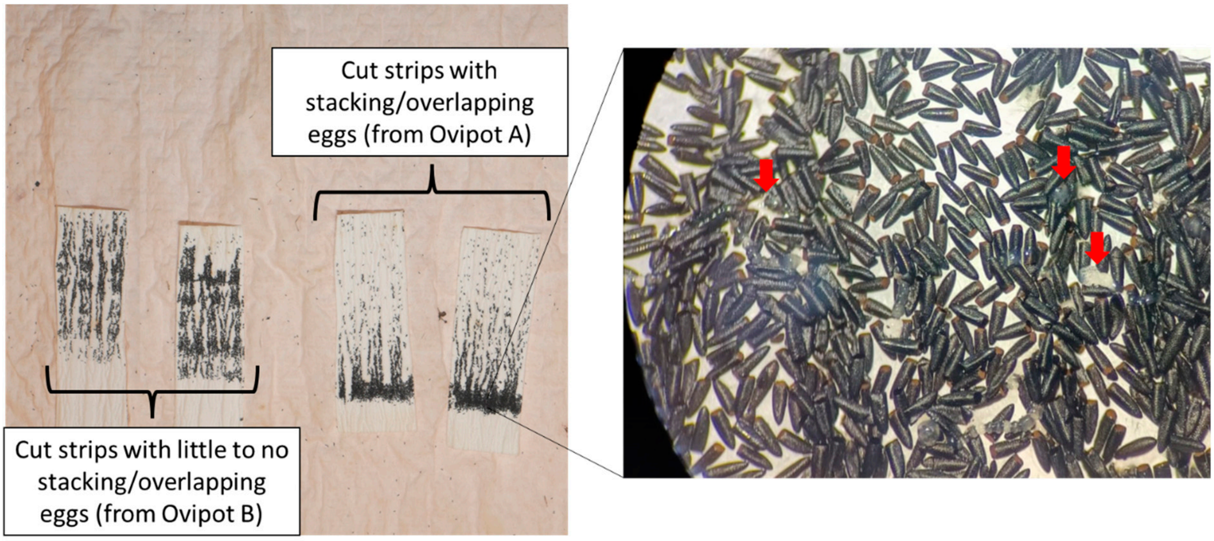

Supplementary Figure S1. Example of cut oviposition papers with stacked eggs (from Ovipot A) and those with little to no stacked eggs (from Ovipot B). The red arrows point to dead L1 larvae among the stacked eggs.

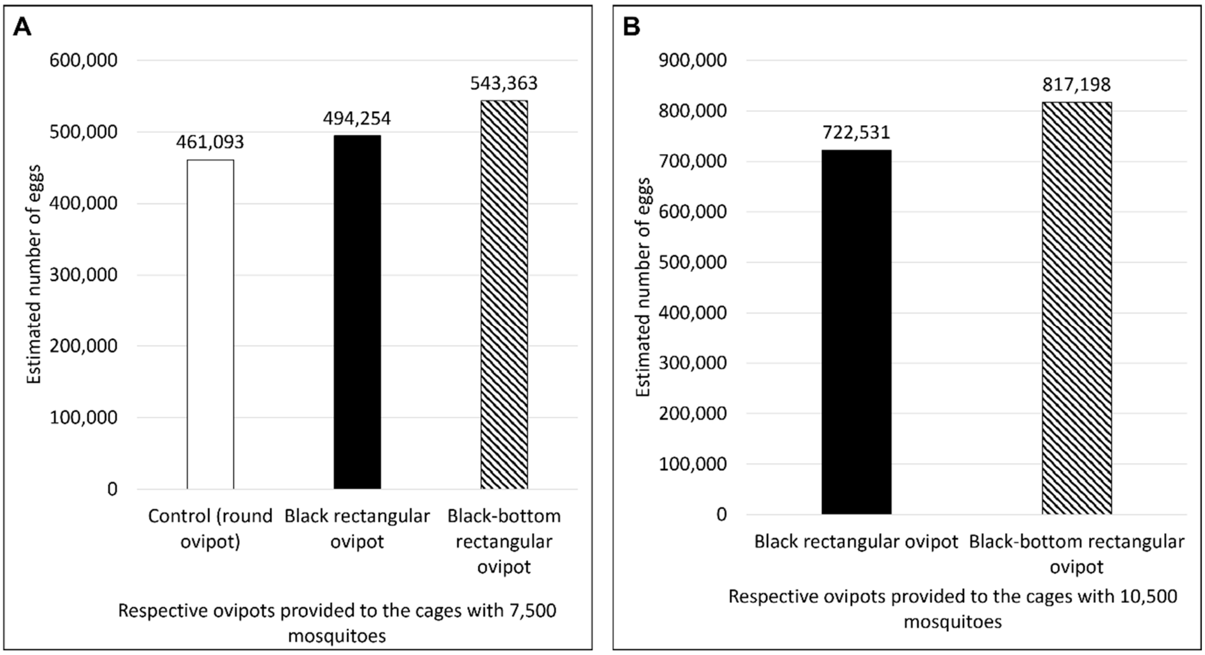

Supplementary Figure S2. (A) Total number of eggs collected across 3 blood meals from the cages (n=1) with 7,500 mosquitoes using different ovipots. **(B)** Average number of eggs collected across 3 blood meals from cages with 10,500 mosquitoes (n=2) using different ovipots.
